# miRNA-155-3p and miRNA-3196 as Potential Biomarkers in Liquid Biopsies of Non-Small Cell Lung Cancer Patients

**DOI:** 10.3390/biomedicines13122946

**Published:** 2025-11-29

**Authors:** Daniela Alexandre, Joana Polido, Salete Valente, Daniel Pimenta Rocha, Alexandra R. Fernandes, Pedro V. Baptista, Carla Cruz

**Affiliations:** 1 RISE-Health, Department of Chemistry, Faculty of Sciences, University of Beira Interior, Rua Marquês d’Ávila e Bolama, 6201-001 Covilhã, Portugal; 2 UCIBIO, Department of Life Sciences, Faculdade de Ciências e Tecnologia, Universidade NOVA de Lisboa, 2829-516 Caparica, Portugal; 3 i4HB, Associate Laboratory—Institute for Health and Bioeconomy, Faculdade de Ciências e Tecnologia, Universidade NOVA de Lisboa, 2829-516 Caparica, Portugal; 4 ULS Cova Beira, Serviço de Pneumologia, 6200-251 Covilhã, Portugal; 5 Departamento de Química, University of Beira Interior, Rua Marquês d’Ávila e Bolama, 6201-001 Covilhã, Portugal

**Keywords:** non-small cell lung cancer, microRNA-155-3p, microRNA-3196, biomarkers, clinicopathologic characteristics, peripheral blood mononuclear cells

## Abstract

**Background/Objectives**: Late diagnosis hampers effective treatment of non-small cell lung cancer (NSCLC). This study evaluated whether circulating microRNAs (miRs), miR-155 and miR-3196, measured in liquid biopsy peripheral blood mononuclear cells (PBMCs), can serve as potential non-invasive biomarkers for NSCLC diagnosis, patient stratification, therapy monitoring, and prognosis. **Methods**: RNA was isolated from PBMCs of 136 NSCLC patients and 64 healthy donors. RT–qPCR quantified miR expression in PBMCs after predefined QC filtering: miR-155-3p (NSCLC *n* = 63; controls *n* = 28), miR-3196 (NSCLC *n* = 55; controls *n* = 28), and miR-155-5p (NSCLC *n* = 23; controls *n* = 12). Diagnostic performance was assessed using receiver operating characteristic (ROC) analyses, reporting area under the curve (AUC), and threshold-dependent sensitivity/specificity. Survival was analyzed with Kaplan–Meier/Cox methods. Associations with clinicopathological variables (stage, metastasis, smoking, EGFR, and KRAS status), treatment response (chemotherapy, immunotherapy, TKIs), and survival outcomes were examined. **Results**: miR-155-3p was upregulated in NSCLC, whereas miR-3196 was downregulated relative to controls; AUCs were 0.881 and 0.784, respectively. At high-sensitivity operating points, specificity was lower (≈29–30%), consistent with PBMC miRs reflecting both immune activation and tumor burden. In adenocarcinoma, miR-155-3p was associated with advanced stage, metastatic disease and smoking history. miR-3196 aligned with features of metastatic progression. During systemic therapy (chemotherapy, immunotherapy, TKIs), circulating levels of both miRs tended to normalize. Notably, normalization of miR-155-3p levels was associated with improved overall survival, supporting its prognostic value and utility for treatment monitoring. **Conclusions**: Circulating miR-155-3p and miR-3196 in PBMCs are promising screening/monitoring non-invasive candidates rather than stand-alone NSCLC diagnostics at current thresholds. Combining these miRs with additional biomarkers and/or clinical covariates and tuning decision thresholds may enhance specificity for diagnostic use. While preliminary, these findings warrant validation in large, prospective studies with standardized protocols to enable clinical implementation.

## 1. Introduction

According to the latest statistics, Lung Cancer (LC) remains a primary global health concern as the most diagnosed cancer and the leading cause of cancer-related deaths worldwide. In 2022, an estimated 2.5 million new cases were reported, representing 12.4% of all cancer diagnoses [1]. Non-small cell lung cancer (NSCLC) constitutes a significantly heterogeneous form of LC, accounting for over 85% of total diagnosed cases and being strongly correlated with tobacco smoking, environmental exposure, and epigenetic changes [2]. The two main subtypes of NSCLC are lung adenocarcinoma (ADC) and lung squamous cell carcinoma (SCC), which have overall 5-year relative survival rates of 17% and 14%, respectively [3]. Globally, lung ADC, the predominant subtype, has a complex genomic landscape and is more common among non-smokers and women [4]. Furthermore, ADC patients can be classified into subgroups based on histological features, genetic mutations, and other factors [5]. Indeed, mutations in genes such as *epidermal growth factor receptor* (*EGFR*), *tumor protein p53* (*TP53*), *Ki-ras2 Kirsten rat sarcoma virus* (*KRAS*), *V-Raf Murine Sarcoma Viral Oncogene Homolog B* (*BRAF*), and *MET*, as well as translocations in *Anaplastic Lymphoma Kinase* (*ALK)*, *ROS proto-oncogene 1* (*ROS1)*, and *RET*, are important for prognosis and therapeutic responses [6]. All these histological and molecular features are critical for tailoring the most effective treatment plan for each patient subgroup, guiding clinical decisions, and improving the prognosis for ADC patients [7].

Current diagnostic methods for NSCLC include a combination of imaging techniques (e.g., X-rays, PET scans, and MRI), tissue biopsies (e.g., bronchoscopy, needle, or surgical biopsies), and molecular testing (e.g., genetic profiling) [8]. Nonetheless, due to the invasive or uneasy nature of these current diagnostic methods and the absence of early-stage diagnostic biomarkers, most NSCLC cases are identified in advanced stages with metastatic spread, reducing the efficacy of therapeutic interventions [9,10]. Hence, further exploring new non-invasive biomarkers, particularly using liquid biopsies (LB), could enhance early detection and disease management, avoiding disease progression.

MicroRNAs (miRs) hold the potential for diagnostic, prognostic, and therapeutic monitoring effectiveness in various cancers, including LC [11,12,13]. miRs are single-stranded and highly conserved non-coding sequences (typically 18–25 nucleotides) that are crucial upstream regulators of gene expression at the post-transcriptional level [14]. Each miR can regulate simultaneously multiple genes, and several distinct miRs can target the same gene. Consequently, a single miR can act as either a tumor suppressor or an oncogene [15]. Indeed, due to this complex interconnection, a miR may function as an oncogene in one type of cancer but as a tumor suppressor in another. These roles will depend on the specific genes that the miRs target [16]. There is no doubt that miRs can be easily identified and isolated from various human body samples, such as tissues, LB, peripheral blood, urine, and sputum [17]. Blood-based miRs biomarkers are particularly attractive due to their excellent stability and the non-invasive advantages of sample collection. Several investigations are underway to define aberrant profiles of single or various sets of miRs in PBMCs. The PBMC gene expression profiles have identified various cancers, including NSCLC, metastatic melanoma, breast, renal, and bladder cancer, distinguishing early- and advanced-stage patients from healthy controls [18,19]. Among the numerous miRs studied, aberrant expressions of miR-155 and miR-3196 have been associated with NSCLC progression through different cellular and molecular pathways [20].

miR-155 is a well-known oncogenic miR frequently upregulated in NSCLC patient samples, particularly in the ADC subtype and those with positive EGFR mutations [21,22,23]. Its overexpression is linked to more aggressive tumors and resistance to radiation and chemotherapy [24,25], and promotes NSCLC cell proliferation and invasion by downregulating SOCS1, SOCS56, and PTEN [23]. However, higher miR-155-5p levels do not strongly correlate with worse survival outcomes [26].

miR-3196, a relatively novel miR, exhibits context-dependent behavior in LC. It is frequently downregulated in LC tissues, suggesting a tumor-suppressive role in the regulation of pro-apoptotic pathways [27]. Due to this dysregulation, miR-3196 has been investigated as a potential diagnostic and prognostic biomarker. It is notably downregulated in patients with EGFR exon 19 deletion and is involved in ADC progression through the MAFG-AS1/miR-3196/SOX12 axis [27]. However, it can be reactivated under therapeutic stress conditions, such as chemotherapy, immunotherapy, or EGFR- tyrosine kinase inhibitors (TKIs), promoting adaptive mechanisms of resistance and cell survival, functionally resembling an oncomiR in scenarios of disease progression or treatments. Xu et al. found that miR-3196 inhibited LC cell apoptosis by downregulating the pro-apoptotic protein p53 upregulated modulator of apoptosis (PUMA) [28]. Collectively, the data indicate that this molecule primarily functions as a tumor suppressor in untreated disease, but it may assume adaptive roles under therapeutic pressure, an important consideration for accurate biomarker interpretation.

To our knowledge, the relationship between the abnormal expression of these miRs and clinicopathological factors, as well as therapeutic response, has not been sufficiently detailed. This study comprehensively analyzed miR-155 and miR-3196 expression levels and their clinical significance in LB from NSCLC patients. By analyzing their expression levels in RNA samples extracted from LB and correlating them with clinicopathological factors and patients’ treatments, we aim to identify predictive and prognostic biomarkers to improve NSCLC diagnosis and treatment management.

## 2. Materials and Methods

### 2.1. Study Participants

This prospective study included 200 samples: 136 LC patients (aged 55 to 93) recruited at the University Hospital Center Cova da Beira between 2020 and 2024, and 64 healthy controls (aged 22 to 62) with no history of malignant pulmonary disorders or other types of cancer. This study followed the ethical standards (Declaration of Helsinki, 1964) and was approved by the Hospital’s Research Ethics Committee (ref: 35/2019). Written informed consent was obtained from all participants before the sample was withdrawn, or any clinical and pathological information was collected from patients’ medical records and pathology reports. Collected clinical data included details such as gender, age, smoking habits (categorized as never-smokers, former smokers, and current smokers), histological LC subtypes (e.g., ADC, SCC, and adenosquamous (ASC)), cancer stage at diagnosis according to Tumor Node Metastasis (TNM) staging system [29] (e.g., Stage I, II, III, IV), and the presence of key genetic mutations (e.g., *EGFR*, *KRAS*, *ALK*, *BRAF*, *Phosphatidylinositol 3-kinase* (*PIK3FA*), *ROS-1*, *RET* and *MET*). Also, treatment details, including surgery, chemotherapy, radiotherapy, targeted therapy, and immunotherapy) were included. The patients’ staging was according to the International Association for the Study of Lung Cancer guidelines [29].

### 2.2. Clinical Samples Collection

The same collection and processing protocol was applied to healthy controls and NSCLC patients. For both groups, five milliliters (mL) of peripheral blood were collected in ethylenediaminetetraacetic acid (EDTA)-containing tubes (Sigma-Aldrich, St. Louis, MO, USA) and immediately centrifuged (2000× *g* for 10 min at 4 °C). The plasma fraction was removed, and the PBMCs isolated using density gradient centrifugation. Briefly, an equivalent volume of 1× phosphate-buffered saline (PBS) was added to the tube, and the mixture was gently mixed by inverting the tube multiple times. Subsequently, the mixture was transferred to a tube containing an equal volume of Pancoll separating solution (PAN-Biotech, GmbH, Aidenbach, Germany) and centrifuged at 936× *g* for 30 min at room temperature. The PBMCs’ “ring” was washed with cold 1× PBS. After two additional washes and centrifugations, the pellet was resuspended in pre-warmed red blood cell lysis solution, gently stirred for 10 min at 37 °C, and then centrifuged at 426× *g* for 10 min at room temperature. The resulting pellet was washed twice with 1× PBS, and finally, the cells were resuspended in 1× PBS and stored at −80 °C until further use.

### 2.3. Gene Expression

The miRNeasy Mini Kit (Qiagen GmbH, Hilden, Germany) was used to extract the total RNA of all samples, including small RNAs, and eluted in 20 µL of RNase-free water. The quality and concentration of the extracted RNA were assessed spectrophotometrically using a NanoDrop ND1000 (Thermo Fisher Scientific Inc., Waltham, MA, USA) at 260 nm, and the samples were stored at −80 °C until further use.

For each sample, total RNA (200 ng in a final volume of 10 µL) was used for reverse transcription using the miRCURY LNA RT Kit (Qiagen GmbH, Hilden, Germany) according to the manufacturer’s instructions. cDNA was diluted 1:30, and an aliquot of 3 µL of diluted cDNA were added to each 10 µL real-time PCR reaction containing 2× miRCURY SYBR^®^ Green Master Mix and the corresponding miRCURY LNA SYBR^®^ Green primers for miR-155-3p, miR-3196 and U6 small nuclear RNA (GeneGlobe IDs: miR-155-3p: YP00204000; miR-3196: YP02119217; U6: YP02119464; all from Qiagen, Hilden, Germany). Reactions were run on a CFX Connected Real-Time PCR Detection System (Bio-Rad Laboratories, Inc., Hercules, CA, USA) using the following cycling conditions: 95 °C for 2 min, followed by 40 cycles of 95 °C for 10 s and 56 °C for 60 s. Relative gene expression levels were determined using the comparative threshold cycle (Ct) method (2^−ΔΔCt^) [30] and using the expression levels of the *U6* gene as reference for normalization purposes.

All qPCRs were performed in technical duplicates. Pre-specified quality control (QC) criteria were applied: (i) replicate pairs were required to have ΔCt ≤ 0.5; reactions with ΔCt > 0.5 were repeated once and excluded if discordance persisted; (ii) melt-curve analysis required a single peak at the expected melting temperature (±0.5 °C); atypical or multiphasic curves were excluded; (iii) U6 stability required Ct within 18–28 with replicate SEM < 0.5; samples outside these limits were excluded for that assay. After QC, statistical outliers were identified within each group (NSCLC, control) using Tukey’s rule and were removed a priori. As a sensitivity check, a robust median absolute deviation (MAD) z-score > 3.5 identified an overlapping set of extreme values. Although all samples yielded sufficient total RNA for miR quantification, a subset of assay-level measurements was excluded under the pre-specified QC/outlier criteria. In addition, miR assays were conducted at different time points as the cohort accrued. Consequently, the inventory of available patient aliquots differed across assays, and not every miR was quantified in every biological sample.

### 2.4. Statistical Analysis

The descriptive data were expressed as the mean ± SEM, and GraphPad software (v8.0.1, Boston, MA, USA) was applied for statistical analysis. The Shapiro–Wilk test was performed on all miR expression data to assess the normality of the distributions. Various statistical methods were used to evaluate the significance of variations in miR expression levels across different groups and subgroups, consistently accounting for the data’s structure and differences in group sizes. In addition, statistical significance was determined using *p* -values; values less than 0.05 were considered significant.

The Mann–Whitney U test, a nonparametric test, was employed to evaluate the significance of differences in miR expression levels between the NSCLC patient and healthy donor groups and between age and gender subgroups within ADC patients. This test is suitable for comparing two independent groups when the data may not follow a normal distribution. To compare miR expression levels across different stages of patients and between metastatic and non-metastatic patients, Student’s *t*-test with Welch’s correction and a one-way ANOVA with Dunnett’s correction were used. Welch’s correction was applied to account for unequal variances between groups in the *t*-test, while Dunnett’s correction in the ANOVA ensured proper control for multiple comparisons when evaluating differences across more than two groups. For mutation subgroup analyses, miR expression was compared across EGFR-, ALK-, KRAS-, BRAF-, PIK3CA-mutated and mutation-negative tumors using the Kruskal–Wallis test, followed, when the global test was significant, by Dunn’s post hoc test for pairwise comparisons between specific mutation categories.

Additionally, a receiver-operator characteristic (ROC) curve was plotted for each miR, and the area under the curve (AUC) was reported with nonparametric 95% confidence intervals (CIs) estimated by the DeLong method. AUC values ranging from 0.700 to 0.799 indicate a reasonably promising candidate biomarker, while those between 0.800 and 0.899 suggest a strong candidate biomarker. An AUC in the range of 0.900 to 1.0 indicates an outstanding candidate biomarker. To explore the sensitivity–specificity trade-offs, we evaluated multiple decision thresholds across the observed marker distribution, reporting for each threshold the sensitivity, specificity, and the derived positive/negative predictive values (PPV/NPV) (assuming the study prevalence) according to standard formulas: PPV = (Se × Prev)/[(Se × Prev) + (1 − Sp) × (1 − Prev)] and NPV = (Sp × (1 − Prev))/[((1 − Se) × Prev) + Sp × (1 − Prev)]. The optimal cut-off was defined by Youden’s J (J = sensitivity + specificity − 1). Analyses of overall survival (OS) were also conducted. OS was defined as the time from the start of treatment to death (regardless of cause of death). Kaplan–Meier curves for OS were constructed, and differences were assessed using the Mantel–Cox (log-rank) test. Hazard ratios (HRs) with 95% CIs were calculated for all analyses [31].

## 3. Results

### 3.1. Comparison of Clinicopathological Characteristics of Study Participants

The main demographic and clinicopathologic characteristics of the 200 participants in the study are summarized in Table 1, namely sex, age, smoking habits, histological type, disease stage, and the presence of specific mutations in relevant genes. The Portuguese study participants comprised 136 NSCLC patients and 64 healthy controls, all Caucasians. The mean age was 69.0 ± 8.5 and 43.0 ± 9.5 years for NSCLC patients and controls, respectively. Among NSCLC patients, 69.1% were male, whereas 78.1% of controls were female. Regarding histological classification, 69.9% of the NSCLC cases were ADC, with a mean age of 69.4 years and consisting of 60 males and 35 females. SCC accounted for 18.4% of cases, with a mean age of 65.8 years, comprising 21 males and 4 females. ASC was observed in 5.1% of patients, with a mean age of 75.8 years, including 6 males and 1 female. The remaining 6.6% of NSCLC cases were classified as rarer subtypes. Furthermore, smoking prevalence among NSCLC patients was 38.2%, with similar rates observed in SCC (40.0%) and ADC (36.8%) subtypes. Most NSCLC patients presented with advanced-stage disease (stage III/IV, 86.5%). Within the NSCLC smoker subgroup, 40.4% did not harbor mutations in the most relevant genes involved in LC pathogenesis.

### 3.2. Comparison of miRs Expression in NSCLC Patients’ Liquid Biopsies

**Figure 1:**
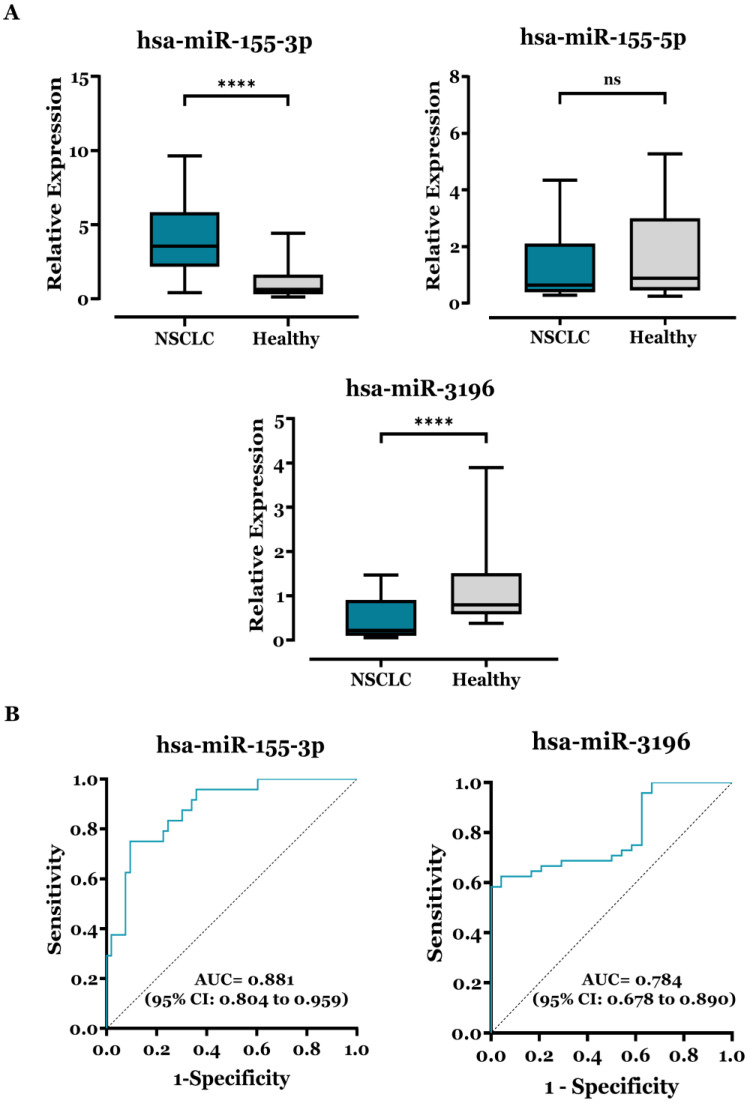
Differential miR expression in PBMCs with ROC curves between NSCLC patients and the healthy donors’ group. (**A**) RT–qPCR analysis of miR-155-3p, miR-155-5p, and miR-3196 expression levels in NSCLC patients and a healthy donors’ group. The expression levels of the three miRs were normalized relative to the corresponding expression level of U6 snRNA, and relative expression was determined using the 2^−ΔΔCt^ method. The Mann–Whitney test was used to assess group differences, and results are expressed as means ± SEM and displayed as box plots. **** *p* < 0.0001 and “ns”: non-significant. Data population after outlier exclusion for multiple miRNAs was: miR-155-3p (NSCLC *n* = 63, controls *n* = 28), miR-155-5p (NSCLC *n* = 23, controls *n* = 12), and miR-3196 (NSCLC *n* = 55, controls *n* = 28). (**B**) The receiver operating characteristic (ROC) curve analysis of PBMC miR-155-3p and miR-3196 for NSCLC detection. AUC (95% CI) are displayed on the curves.

The relative expression of miR-155 and miR-3196 in the LB (PBMC samples) from NSCLC patients and healthy controls was evaluated by RT-qPCR. As shown in Figure 1A the Mann–Whitney test revealed that miR-155-3p expression was significantly upregulated. In contrast, miR-3196 expression was significantly downregulated in the NSCLC group, with mean expression levels of 4.10 ± 0.35 and 0.49 ± 0.07, respectively, compared with the control group, which exhibited mean expression levels of 1.11 ± 0.23 and 1.13 ± 0.18, respectively. In contrast, miR-155-5p did not show a statistically significant difference in expression between the two groups. Figure S1 provides box plots with outliers highlighted.

To evaluate the diagnostic performance of the two candidates, ROC curve analysis was performed for miR-155-3p and miR-3196 in PBMCs (Figure 1B). miR-155-3p achieved an AUC of 0.881, consistent with strong discriminative ability, whereas miR-3196 exhibited an AUC of 0.784, indicating a promising but less powerful marker.

Threshold analysis highlighted a clear sensitivity–specificity trade-off for both miRs (Table S1; Figure S2). For miR-155-3p, all thresholds up to approximately 0.39 ΔΔCt retained 100% sensitivity but showed very low specificity, ranging only from 4.2% to 29.2%, indicating a high false-positive rate among healthy controls. Increasing the decision threshold progressively reduced sensitivity while improving specificity, with a Youden-optimized cut-off around 1.13 ΔΔCt providing a more balanced profile (sensitivity 90.6%, specificity 75.0%, PPV 90.6% and NPV 78.9%; Table S1). This pattern confirms that very low thresholds are suitable only for maximally sensitive screening, whereas higher thresholds are required for more specific diagnostic use. For miR-3196, the opposite tendency was observed: very low thresholds (e.g., <0.08 ΔΔCt) yielded 100% specificity but very poor sensitivity (≈2–17%), whereas intermediate thresholds around 0.37–0.39 ΔΔCt improved sensitivity to approximately 58% while maintaining high specificity (95–100%), with corresponding PPV and NPV in the 52–58% range (Table S1). Thus, miR-3196 behaves as a marker with intrinsically high specificity but only moderate sensitivity, and its diagnostic performance is optimized at high-specificity operating points.

### 3.3. Association Between miR Expression and Clinicopathological Parameters of NSCLC Patients

To investigate potential association between the expression levels of miR-155-3p and miR-3196 and clinicopathological subgroups of NSCLC patients, we focused exclusively on patients with the ADC histological subtype, comprising 95 patients (see Table 1). Accordingly, we analyzed the associations between miR expression levels and various clinicopathological parameters of ADC patients, including tumor stage, metastatic state, age, gender, genetic mutations, and smoking history. The comparison analysis is summarized in Table S2.

**Figure 2:**
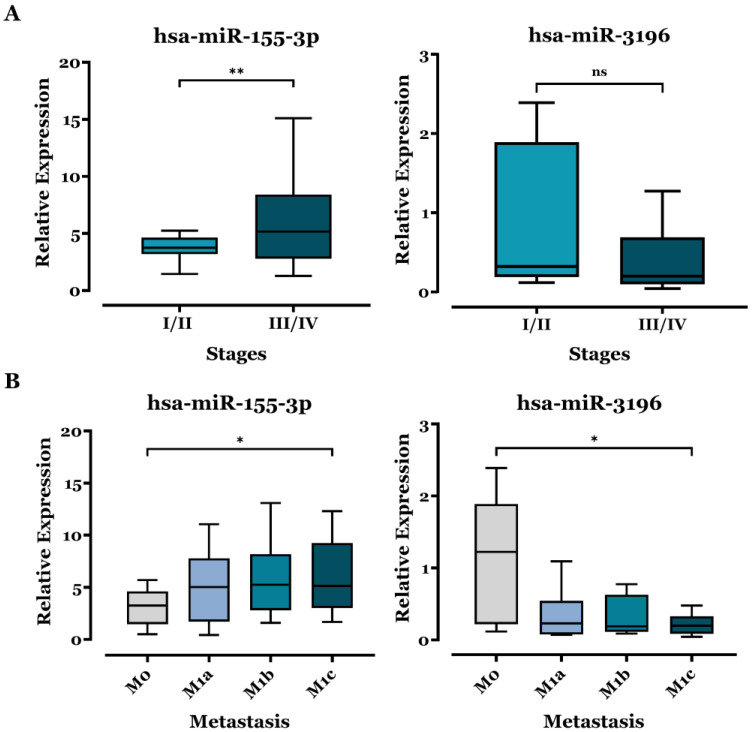
Comparison of miR-155-3p and miR-3196 expression in different subgroups of ADC patients. (**A**) RT–qPCR analysis of differential miRs levels in different clinical stages of patients. The expression levels of miRs were normalized relative to the corresponding expression level of U6 snRNA, and relative expression was determined using the 2^−ΔΔCt^ method. Student’s *t*-test with Welch’s correction was used to assess significance, and results are expressed as means ± SEM and displayed as box plots. ** *p* <  0.01 and “ns”: non-significant. (**B**) RT–qPCR analysis of miR levels in different metastasis subgroups of patients. The expression levels of miRs were normalized relative to the corresponding expression level of U6 snRNA, and relative expression was determined using the 2^−ΔΔCt^ method. The one-way ANOVA with Dunnett’s correction was used to assess group differences, and results are presented as means ± SEM and displayed as box plots. * *p* < 0.05.

Figure 2 illustrates the relative expression levels of miR-155-3p and miR-3196 across different tumor stages (Figure 2A) and metastatic states (Figure 2B). For the tumor stage, the mean expression level of miR-155-3p was significantly higher in advanced stages (III/IV: 5.90 ± 0.64) compared to early stages (I/II: 3.69 ± 0.46), as determined by Student’s *t*-test, indicating an association between miR-155-3p up-regulation in LB and tumor progression. Conversely, although miR-3196 expression decreased with advancing stages, the difference between early (I/II: 0.91 ± 0.40) and advanced stages (III/IV: 0.41 ± 0.09) was not statistically significant. Nevertheless, if more patients were enrolled in each subgroup, this relation could be clarified.

When comparing expression levels across metastatic states, miR-155-3p levels progressively increased with the degree of metastasis (M0 to M1c), showing statistically significant upregulation in the M1c group (mean of 6.12 ± 1.01) compared to the M0 group (mean of 3.13 ± 0.54). Additionally, miR-3196 expression levels were significantly reduced in metastatic states, decreasing from the M0 group (mean of 1.17 ± 0.33) to the M1c group (mean of 0.21 ± 0.05), as depicted in Figure 2B.

**Figure 3:**
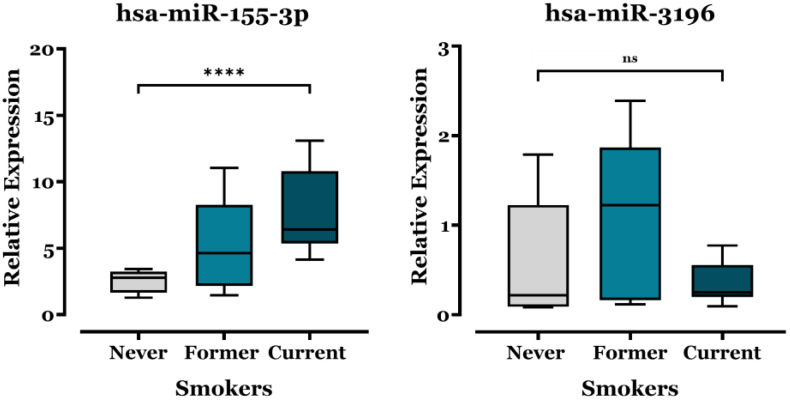
Comparison of miR-155-3p and miR-3196 expression in the different smoking histories of ADC patients. RT–qPCR analysis of the difference in miR-155-3p and miR-3196 levels in different smoking histories of patients. The expression levels of miRs were normalized relative to the corresponding expression level of U6 snRNA, and relative expression was determined using the 2^−ΔΔCt^ method. The one-way ANOVA with Dunnett’s correction was used to assess group differences, and values are expressed as means ± SEM and displayed as box plots. **** *p* <  0.0001 and “ns”: non-significant.

We also investigated the potential association between dysregulated miR expression and the patients’ smoking habits. The study group was categorized into three subgroups: non-smokers (individuals with no history of active smoking), former smokers (individuals who had abstained from smoking for at least the last five years), and current smokers. Results revealed a significant association between miR-155-3p expression levels and smoking history, as shown in Figure 3. Specifically, miR-155-3p expression was significantly higher in current smokers (mean of 7.70 ± 0.73) than non-smokers (mean of 2.58 ± 0.25). This indicates a potential association between active smoking and the upregulation of miR-155-3p in ADC patients. On the contrary, the expression levels of miR-3196 did not show any significant differences across the smoking subgroups, suggesting that miR-3196 expression is independent of smoking history in ADC patients.

Currently, limited information is available in the literature on the expression levels of miR-155-3p and miR-3196 in ADC patients with mutations in critical oncogenic driver genes. In our study, we also investigated whether genetic alterations in genes associated with LC carcinogenesis correlated with the expression levels of these two miRs.

**Figure 4:**
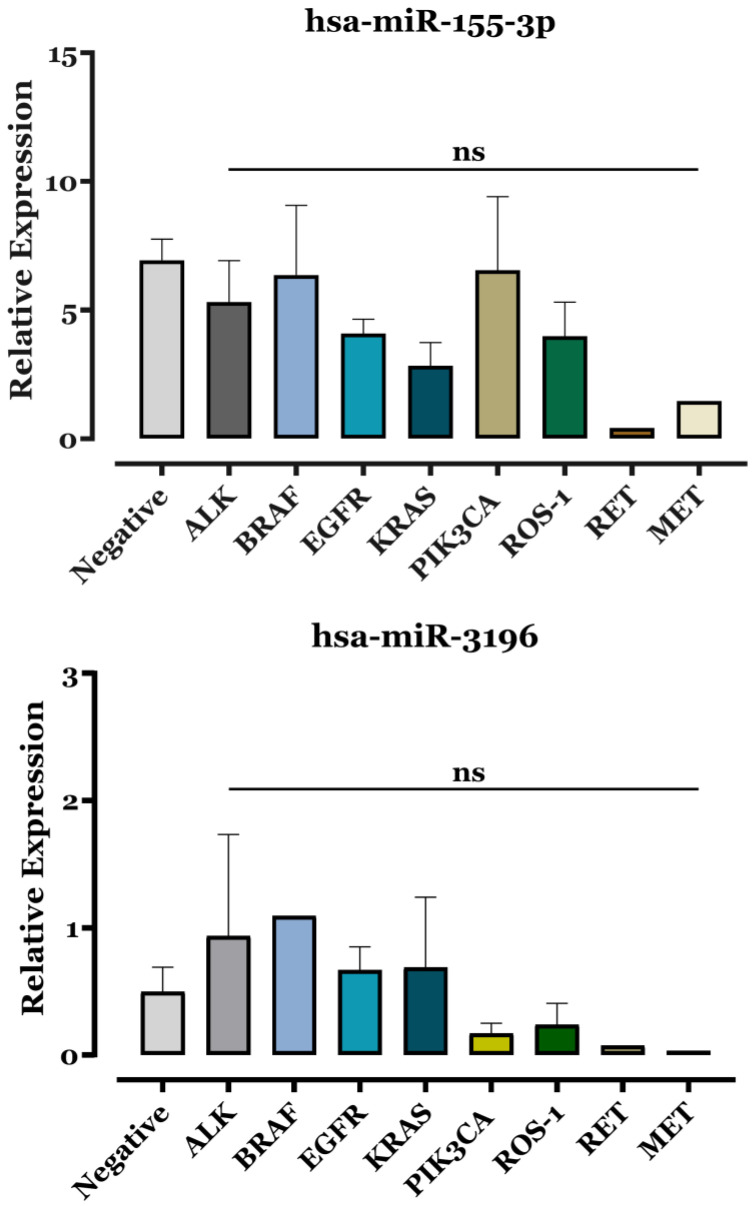
Comparison of miR-155-3p and miR-3196 expression in different gene mutations of ADC patients. RT–qPCR analysis of the differential in miR-155-3p and miR-3196 levels in different gene mutations. The expression levels of miRs were normalized relative to the corresponding expression level of U6 snRNA, and relative expression was determined using the 2^−ΔΔCt^ method. Kruskal–Wallis testing with Dunn’s comparisons was used to assess group differences, and results are expressed as means ± SEM and displayed as bars. No comparison reached statistical significance, and all differences are indicated as non-significant (“ns”).

According to Figure 4, no overall differences in miR-155-3p or miR-3196 expression were detected among mutation groups by Kruskal–Wallis testing (*p* > 0.05). Pairwise Dunn’s comparisons between individual mutation categories (*EGFR*, *KRAS*, *ALK*, *BRAF*, *PIK3CA*, *ROS1*) and the mutation-negative group were likewise non-significant (“ns”), indicating that, within the limits of our sample size, these oncogenic alterations do not exert a detectable effect on PBMC miR-155-3p or miR-3196 levels. Although some groups (e.g., *EGFR*- and *KRAS*-mutated ADCs, with mean miR-155-3p values of 4.08 ± 0.56 and 2.84 ± 0.89, respectively) showed numerical trends towards different mean miR-155-3p values, these differences did not reach statistical significance and should therefore be interpreted with caution. These findings suggest that miR-155-3p may play a role in specific oncogenic pathways, particularly those involving *EGFR* and *KRAS* mutations.

Finally, no statistically significant associations were detected between the expression levels of both miRs and the age of ADC patients (Figure S3). However, miR-3196 levels have been shown to correlate with patient gender, with a significant difference observed between males (mean 1.03 ± 0.19) and females (mean 0.41 ± 0.11).

### 3.4. Association of miR Expression with Treatments and Survival Outcomes

Studying changes in miR-155-3p and miR-3196 expression levels in response to various treatments offers valuable insights into the mechanisms underlying treatment efficacy and disease progression. Hence, to evaluate their potential as biomarkers for predicting treatment responses in ADC patients, we compared their expression levels between untreated individuals and those undergoing various treatments, including surgery, chemotherapy, immunotherapy, radiotherapy, and TKIs. Treatment order and application frequency were not considered in this study.

**Figure 5:**
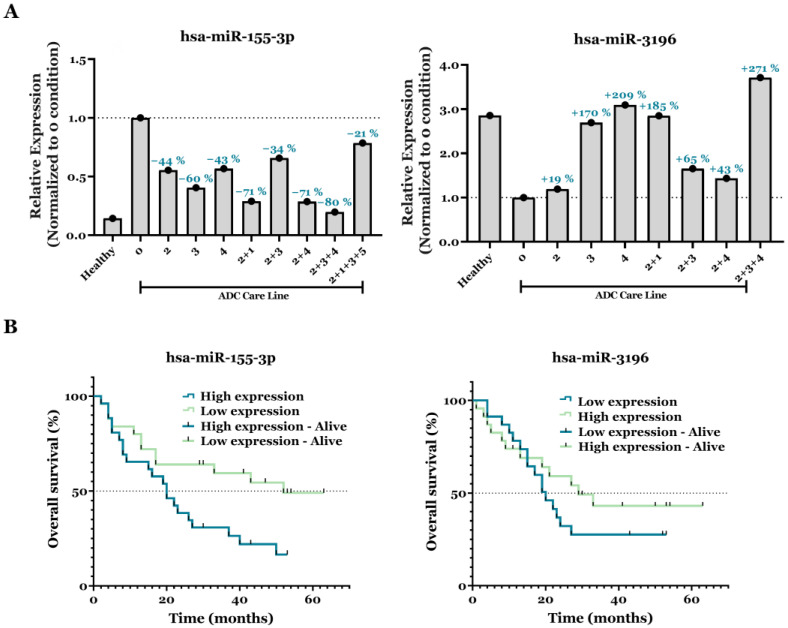
The impact of different lines of therapy on miRs expression levels and their association with overall survival. (**A**) Comparative RT–qPCR analysis of basal and post-therapeutic expression levels of miR-155-3p and miR-3196 in ADC patients. Expression levels relative to U6 snRNA were assessed using the 2^−ΔΔCt^ method, and all data were further normalized to ADC patients without any treatment (0 condition). The values are expressed in means and displayed on a scatter graph with bars. The dotted horizontal line at 1.0 indicates the normalization reference (expression equal to the 0 condition). Treatment codes: 1 = Surgery, 2 = Chemotherapy, 3 = Immunotherapy, 4 = Tyrosine kinase inhibitors (TKIs), 5 = Radiotherapy. (**B**) Kaplan–Meier curves illustrate the potential of miR-155-3p and miR-3196 expression levels as biomarkers for predicting ADC patient survival. The miR-155-3p and miR-3196 were dichotomized based on low (2^−ΔΔCt^ < 3.7663 and <0.2709, respectively) versus high expression (2^−ΔΔCt^ ≥ 3.7663 and ≥0.2709, respectively). The dotted horizontal line indicates 50% overall survival.

Figure 5A illustrates the relative expression levels of miR-155-3p and miR-3196, normalized to untreated patients (baseline or “0 condition”), with percentage changes indicated above each bar. Our findings reveal consistent downregulation of miR-155-3p across all treatment groups relative to the 0 condition, suggesting its suppression in response to therapy and a possible role in regulatory pathways. The most significant decrease (80%) occurred in patients receiving chemotherapy, immunotherapy, and inhibitors (2 + 3 + 4 condition), reducing miR-155-3p expression to levels comparable to those of healthy individuals. Other treatment groups showed moderate downregulation, ranging from 21% to 71%. Conversely, miR-3196 showed a positive regulation across all ADC care line groups. The largest increase (271%) was observed in patients treated with chemotherapy, immunotherapy, and inhibitors (2 + 3 + 4 condition), mirroring the trend observed with miR-155-3p downregulation. This suggests that these treatments strongly induce miR-3196, potentially playing an active role in the biological response to therapy. Other treatment groups also showed substantial increases in miR-3196 expression, ranging from 19% to 209%. These findings highlight miR-3196 as a potential therapeutic biomarker and reinforce the differential regulatory roles of both miRs in ADC treatment response.

To further assess the impact of miRs on clinical outcomes, survival analysis was performed using Kaplan–Meier curves and Cox regression. Regarding OS, the median follow-up for the entire cohort was 22 months (range 1–63 months). In Figure 5B, the survival curves analysis revealed that high miR-155-3p expression was significantly associated with shorter OS than lower expression levels (median OS: 20 months vs. 52 months; HR = 2.31; 95% CI, 1.14–4.68; *p*-value = 0.0196). Thus, miR-155-3p appears to be a negative prognostic biomarker, and its reduction after treatment may correlate with better survival. Conversely, increased miR-3196 expression did not demonstrate a significant association with improved OS (median OS: 29 months vs. 20 months; HR = 1.45; 95% CI, 0.68–3.08; *p*-value = 0.3364). Therefore, modulating miR-3196 may not reliably improve outcomes based on current evidence.

## 4. Discussion

Notably, LC remains a significant public health challenge in Portugal. In 2022, 6155 new cases (8.8%) were diagnosed, and 5077 deaths (15%) were recorded, making it the third most common cancer and the leading cause of cancer-related mortality in Portugal [1]. Survival rates are critically low, with only 15.1% of patients living beyond five years—slightly above the European average (13%) but trailing behind the U.S. (19%) [32]. The gender disparity is stark, with incidence rates significantly higher in males (36.9 per 100,000) compared to females (13.3 per 100,000) [1]. These numbers highlight an urgent need for stronger early detection programs and more effective treatment strategies to combat the devastating impact of LC in Portugal and beyond.

miRs have emerged as robust biomarkers due to their strong association with various cancers, making them valuable for early detection and treatment monitoring [33,34]. While miRs analysis in tissue samples has been widely used for tumor prognosis studies, LB offers key advantages: it is non-invasive, cost-effective, portable, and allows for real-time monitoring of survival prognosis before or after treatment [26]. NSCLC is particularly linked to widespread miRs dysregulation, affecting multiple mRNA targets. Among them, miR-155 and miR-3196 have shown strong potential as biomarkers. Consequently, this study aims to explore their diagnostic, prognostic, and therapeutic monitoring value by analyzing their expression profiles in PBMCs from LB, identifying clinically relevant patterns across different patient conditions.

miR-155 is a key regulator of cellular processes and one of the earliest miRs identified as dysregulated in many diseases [34]. Both miR-155-3p and its counterpart miR-155-5p are implicated in immune modulation and tumor development, with dysregulated expression linked to LC [35,36,37]. Elevated miR-155 levels in tissues, plasma, and sputum are associated with a higher risk of NSCLC, reinforcing its oncogenic potential, though its ability in predicting disease progression remains unclear [20,26]. Notably, miR-155-5p correlates with tumor subtypes, poor survival, and clinicopathologic markers, making it a promising diagnostic biomarker, whereas miR-155-3p’s role in LC remains largely unexplored.

Our findings reveal significantly elevated miR-155-3p expression in LB from NSCLC patients compared to healthy controls (Figure 1), challenging prior reports of miR-155 downregulation [20]. This discrepancy likely stems from differences in patient cohorts and experimental conditions. Unlike miR-155-3p, miR-155-5p showed no significant dysregulation. Nevertheless, the fluctuating miR-155-5p/-3p ratio, influenced by immune response timing and regulatory interactions, may explain this discrepancy [26]. Additionally, ROC curve analysis confirmed that miR-155-3p has good overall discriminative performance (AUC = 0.881), but its operating characteristics depend strongly on the chosen threshold [26]. At the lowest ΔΔCt cut-offs, sensitivity approaches 100%, yet specificity is very poor (4.2–29.2%), implying a substantial fraction of false-positive classifications among healthy controls (Table S1). As the threshold increases, sensitivity decreases while specificity improves, with an intermediate cut-off around 1.13 ΔΔCt providing a more balanced profile (sensitivity 90.6%, specificity 75.0%, PPV 90.6% and NPV 78.9%). This behavior is biologically plausible, as PBMC miRs are expected to capture systemic immune activation and inflammation in addition to tumor-derived signals, and miR-155 is well documented to be upregulated in smokers and in inflammatory lung conditions. Consequently, PBMC miR-155-3p should not be viewed as a tumor-exclusive signal but rather as a composite readout of both cancer burden and host immune status.

miR-155-3p levels were particularly elevated in advanced NSCLC stages (III–IV) and aggressive metastasis subgroups (M1c) of ADC patients (Figure 2), linking its expression to tumor progression and metastasis, as with miR-155-5p [38]. Moreover, smoking history correlated with increased miR-155-3p expression (Figure 3), suggesting smoking may influence its regulation and potentially contribute to NSCLC development. This aligns with findings by De Smet et al., who reported higher miR-155 levels in lung tissue from smokers and patients with lung inflammation [39]. In other contexts, circulating serum miR-155 levels also increase in healthy individuals after smoking a single cigarette [40]. Further analysis (Figure S2) showed no significant differences in miR-155-3p expression based on age or gender. However, expression levels tended to be lower in KRAS- and EGFR-mutated tumors (Figure 4), suggesting a possible but as yet non-significant association with oncogenic pathways specific to these mutations. Supporting this, Dacic et al. found that miR-155 was upregulated only in EGFR/KRAS-negative ADC patients, reinforcing our findings [41].

Importantly, our study highlights miR-155-3p as a potential predictive biomarker for treatment response (Figure 5). Nevertheless, it is important to note that treatment effects on miRs expression vary, making the timing of post-treatment measurements crucial. Our results suggest that miR-155-3p is involved in treatment-regulated pathways, with the most pronounced decrease (80%) observed in patients receiving combined chemotherapy, immunotherapy, and TKIs. As chemotherapeutic agents induce cellular stress and DNA damage, they can alter miR-155 expression [42], sometimes promoting chemoresistance [25,43]. However, we observed downregulation of miR-155-3p in patients receiving combination therapy, underscoring the complex relationship between chemotherapy and miRs within the tumor microenvironment. Immunotherapy, which modulates immune cell activity, can indirectly affect the tumor microenvironment and gene expression, including miR-155, which plays a key role in immune cell function [44]. While some studies suggest immunotherapy increases miR-155 levels, our results show a decrease, possibly influenced by therapy type, cancer subtype, and individual immune response. Similarly, inhibitor therapies, particularly targeted therapies, aim to block specific signaling pathways crucial for cancer cell growth [45]. While TKIs do not directly target miR-155 by inhibiting specific tyrosine kinases, TKIs can disrupt downstream signaling pathways that may indirectly affect miR-155-3p expression [46]. This mechanism supports the decreased levels observed in patients treated with TKIs in our study.

Patients’ Kaplan–Meier curve analysis also strongly supports the clinical relevance of miR-155-3p dynamics. A reduction in miR-155-3p expression post-treatment correlates with favorable clinical outcomes and a lower risk of death, suggesting that miRs may actively contribute to maintaining minimal residual disease and/or long-term remission. These findings position miR-155-3p as a critical regulator of disease dynamics and a promising therapeutic target. Interventions that directly or indirectly suppress miR-155-3p expression may enhance the depth and durability of response, potentially reducing relapse rates.

The p53-responsive miR-3196 has been identified as a tumor suppressor in different cancers, though research remains limited [47]. Experimental evidence suggests it regulates key oncogenic pathways involved in cell proliferation, invasion, metastasis, and apoptosis [28,48]. The first report linking LC to miR-3196 was by C. Xu et al. in 2016, demonstrating significant downregulation in LC tissues and cell lines compared to controls [28].

In this study, our findings (see Figure 1) align with previous research confirming that miR-3196 is downregulated in NSCLC patients compared to healthy controls. From a diagnostic standpoint, miR-3196 displayed an AUC of 0.784, consistent with a moderately strong biomarker, but its threshold behavior differed from that of miR-155-3p. Very low ΔΔCt thresholds yielded 100% specificity but extremely low sensitivity (2–17%), indicating that only a small fraction of NSCLC patients would be detected at these cut-offs (Table S1). As the threshold was relaxed towards intermediate values (≈0.35–0.39 ΔΔCt), sensitivity increased to approximately 58%, while specificity remained very high (95–100%), yielding Youden indices in the 0.54–0.58 range. Thus, miR-3196 essentially acts as a high-specificity, moderate-sensitivity marker, which may be more useful as a confirmatory or complementary component within a multimarker panel than as a stand-alone screening test.

In gastric cancer, miR-3196 has been linked to lymph node metastasis and TNM stage [49]. In this sense, to explore similar associations in LC, we analyzed its expression across different NSCLC stages. However, no significant differences were observed (Figure 2). This outcome might have reached statistical significance with a larger sample size, aligning with the above-mentioned observations for other samples and cancer types. Interestingly, in ADC patients, miR-3196 expression was notably lower in metastatic cases, suggesting a potential role in metastasis suppression or its loss as a hallmark of disease progression. We further examined miR-3196 levels in relation to age, gender, and gene mutations in ADC patients (Figures S2 and 4). While previous studies reported downregulation of ADC in patients with EGFR mutations compared with wild-type [27], our results revealed no significant differences across genetic subtypes. This challenges prior findings and suggests that oncogenic mutations may not strongly influence miR-3196 expression.

Lastly, our study highlights miR-3196 as a promising biomarker for predicting treatment response (Figure 5). Similarly to previously studied miR-155, miR-3196 expression is influenced by various therapies, with timing a critical factor given its dynamic changes. Notably, miR-3196 levels increased significantly, peaking at 271% in patients receiving combined chemotherapy, immunotherapy, and TKIs. While chemotherapy alone had no significant effect, immunotherapy and TKIs individually increased miR-3196 expression levels. The pronounced effect of combination therapy likely stems from immunotherapy’s immune-regulatory influence and TKIs’ disruption of key signaling pathways critical for miRs regulation. These findings underscore the importance of understanding the molecular mechanisms driving miRs modulation in response to specific treatment options. Meanwhile, although the increase in miR-3196 expression after post-treatment showed a non-significant trend toward better survival, its modulation was not clearly associated with prolonged survival in this cohort.

Taken together, these observations indicate that PBMC miR-155-3p and miR-3196 should primarily be regarded as promising non-invasive screening and monitoring candidates rather than stand-alone diagnostic tests at the current thresholds. miR-155-3p offers high overall sensitivity but limited specificity at very low cut-offs, whereas miR-3196 contributes complementary, high-specificity information at the cost of lower sensitivity. A pragmatic clinical implementation would therefore involve a two-step strategy: a high-sensitivity LB screen followed by confirmatory imaging and/or additional molecular testing. Future studies should evaluate whether combining these miRs with other miRs and clinical parameters can achieve higher specificity for diagnostic decision-making.

## 5. Conclusions

This study highlights the potential of miR-155-3p and miR-3196 as promising non-invasive biomarkers that may support NSCLC screening, risk stratification, and treatment monitoring, rather than serving as stand-alone diagnostic tests at current thresholds. We confirmed that miR-155-3p is significantly upregulated in NSCLC patients and associates with advanced stage, metastasis, and smoking history. Conversely, miR-3196 is downregulated, with lower expression levels associated with metastatic progression. Notably, both miR levels showed therapy-related modulation, with partial normalization in patients receiving combined chemotherapy, immunotherapy, and TKIs, underscoring their utility as dynamic indicators of systemic treatment response. Furthermore, post-treatment reduction in miR-155-3p was associated with sustained disease control and improved survival in ADC patients, suggesting a dual role for this miR as a therapeutic target and a prognostic biomarker of minimal residual disease.

In summary, integrating these two miRs into clinical practice could enhance early detection efforts and facilitate more personalized treatment strategies, ultimately improving patient outcomes. The alignment of these findings with the existing literature further supports the robustness of our methodology. Nevertheless, certain limitations should be considered. Our relatively small sample size may affect the generalizability of the results, and the lack of standardized measurement protocols poses challenges for clinical translation. Future large-scale prospective studies are needed to validate these biomarkers and optimize their implementation in diagnostic and therapeutic workflows.

## Figures and Tables

**Table 1:** Basic clinicopathologic characteristics of NSCLC patients and healthy controls.

Characteristic			NSCLC Patients (*n* = 136)	Healthy Donors (*n* = 64)
**Individuals parameters**	Gender *n*, (%)	Male	94 (69.1%)	14 (21.9%)
		Female	42 (30.9%)	50 (78.1%)
	Age (mean ± SD, years)		69 ± 9	43 ± 11
	Smoking status *n*, (%)	Never smoker	45 (33.1%)	41 (64.1%)
		Former smoker	39 (28.7%)	8 (12.5%)
		Current smoker	52 (38.2%)	15 (23.4%)
**Tumors parameters**	Histological type *n*, (%)	ADC	95 (69.9%)	N/A
		SCC	25 (18.4%)	
		ASC	7 (5.1%)	
		Other	9 (6.6%)	
	TNM stage *n*, (%)	I/II	22 (16.2%)	N/A
		III/IV	114 (83.2)	
	Metastasis (M factor)	Mx	2 (1.4%)	
		M0	41 (30.2%)	N/A
		M1a	33 (24.3%)	
		M1b	18 (13.2%)	
		M1c	42 (30.9%)	
	Gene Mutations *n*, (%)	Without	68 (50%)	N/A
		With	58 (42.6%)	
		Not Available	10 (7.4%)	N/A

## Data Availability

The original contributions presented in this study are included in the article. Further inquiries can be directed to the corresponding authors.

## Supplementary Materials

The following supporting information can be downloaded at https://www.mdpi.com/article/10.3390/biomedicines13122946/s1. Figure S1: Differential miR expression in PBMCs between NSCLC patients and the healthy donors’ group. Table S1: Sensitivity. Specificity. Positive predictive value (PPV). Negative predictive value (NPV) for miR-155-3p and miR-3196 (with 95% CIs) across a grid of ΔΔCt thresholds; Figure S2: Sensitivity and specificity as a function of the decision threshold for PBMC miR-155-3p and miR-3196; Figure S3: Comparison of miR-155-3p and miR-3196 expression in different subgroups of ADC patients; Table S2: The comparison between miR-155-3p and miR-3196 levels and clinicopathological parameters in NSCLC patients.

## Abbreviations

The following abbreviations are used in this manuscript:

°C	Celsius
ADC	Adenocarcinoma
ALK	Anaplastic Lymphoma Kinase
ANOVA	Analysis of Variance
ASC	Adenosquamous Carcinoma
AUC	Area Under the Curve
BRAF	v-Raf murine sarcoma viral oncogene homolog B1
CI	Confidence Interval
Ct	Threshold cycle
EDTA	Ethylenediaminetetraacetic Acid
EGFR	Epidermal Growth Factor Receptor
HR	Hazard Ratio
KM	Kaplan–Meier
KRAS	Ki-ras2 Kirsten rat sarcoma viral oncogene homolog
LB	Liquid Biopsy
LC	Lung Cancer
LNA	Locked Nucleic Acid
MAFG-AS1	MAFG Antisense RNA 1
MET	MET proto-oncogene, receptor tyrosine kinase
miR	microRNA
mL	milliliters
MRI	Magnetic Resonance Imaging
NSCLC	Non-Small Cell Lung Cancer
OS	Overall Survival
PBMC	Peripheral Blood Mononuclear Cells
PBS	Phosphate-Buffered Saline
PET	Positron Emission Tomography
PIK3CA	Phosphatidylinositol-4,5-bisphosphate 3-kinase catalytic subunit alpha
PTEN	Phosphatase and TENsin homolog
PUMA	p53-Upregulated Modulator of Apoptosis
QC	Quality control
ROC	Receiver Operating Characteristic
ROS-1	ROS proto-oncogene 1
RT-qPCR	Reverse Transcription quantitative Polymerase Chain Reaction
SCC	Squamous Cell Carcinoma
SEM	Standard Error of the Mean
snRNA	small nuclear RNA
SOCS1	Suppressor of Cytokine Signaling 1
SOCS6	Suppressor of Cytokine Signaling 6
SOX12	SRY-Box Transcription Factor 12
TKIs	Tyrosine Kinase Inhibitors
TNM	Tumor-Node-Metastasis staging system
TP53	Tumor Protein p53
U6	U6 small nuclear RNA
