# Supplementary material for: miRNA-155-3p and miRNA-3196 as Potential Biomarkers in Liquid Biopsies of Non-Small Cell Lung Cancer Patients
Source: Biomedicines. 2025 Nov 29;13(12):2946. doi: 10.3390/biomedicines13122946 (PMC12730197; doi:10.3390/biomedicines13122946)
Supplement: Supplementary file 1 [file biomedicines-13-02946-s001.zip › biomedicines-3962105-supplementary.pdf]

Supplementary Information

miRNA-155-3p and miRNA-3196 as Potential Biomarkers in Liquid Biopsies of Non-Small Cell Lung Cancer Patients

Daniela Alexandre [1,2,3], Joana Polido [1], Salete Valente [4], Daniel Pimenta Rocha [4], Alexandra R. Fernandes [2,3], Pedro V. Baptista [2,3,*] and Carla Cruz [1,5,*]

[1] RISE-Health, Department of Chemistry, Faculty of Sciences, University of Beira Interior, Rua Marquês d'Ávila e Bolama, 6201-001 Covilhã, Portugal
[2] UCIBIO, Department of Life Sciences, Faculdade de Ciências e Tecnologia, Universidade NOVA de Lisboa, 2829-516 Caparica, Portugal
[3] i4HB, Associate Laboratory—Institute for Health and Bioeconomy, Faculdade de Ciências e Tecnologia, Universidade NOVA de Lisboa, 2829-516 Caparica, Portugal
[4] ULS Cova Beira, Serviço de Pneumologia, 6200-251 Covilhã, Portugal
[5] Departamento de Química, University of Beira Interior, Rua Marquês d'Ávila e Bolama, 6201-001 Covilhã, Portugal
*Correspondence: pmvb@fct.unl.pt (P.V.B.); carlacruz@fcsaude.ubi.pt (C.C.)

A

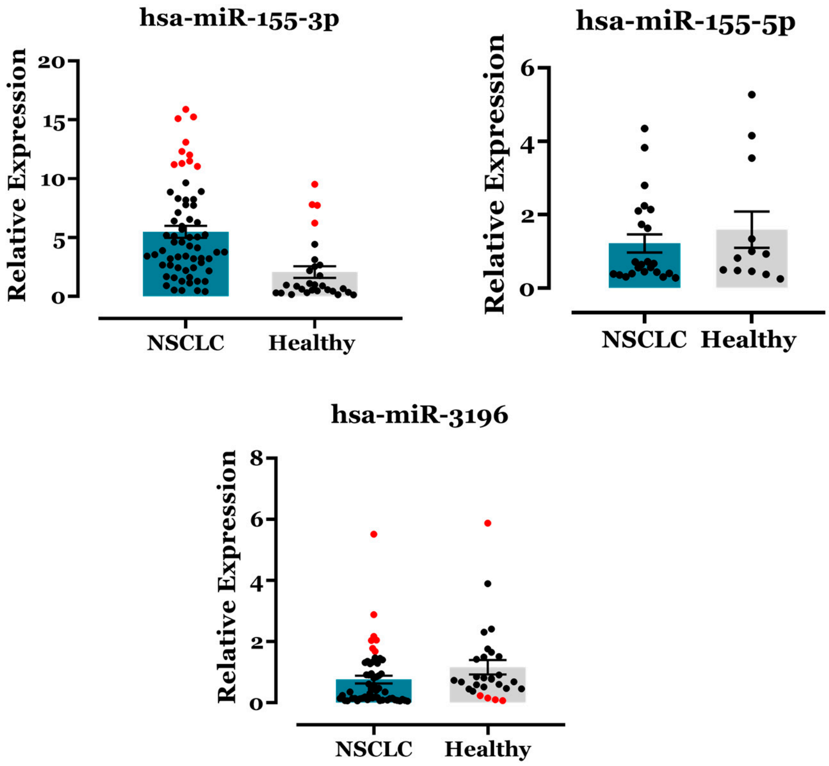

Figure S1. Differential miR expression in PBMCs between NSCLC patients and the healthy donors' group. RT–qPCR analysis of miR-155-3p, miR-155-5p, and miR-3196 expression levels in NSCLC patients and a healthy donors' group. The expression levels of the three miRs were normalized relative to the corresponding expression level of U6 snRNA, and relative expression was determined using the $2^{-\Delta\Delta Ct}$ method. Plots show individual values overlaid on box-and-whisker plots. The bars indicate mean ± SEM. Outliers are highlighted in red and were excluded from inferential testing according to the predefined QC criteria.

Table S1. Sensitivity, specificity, positive predictive value (PPV), and negative predictive value (NPV) for miR-155-3p and miR-3196 (with 95% CIs) across a grid of ΔΔCt thresholds.

miR	Threshold (ΔΔCt)	Sensitivity	95% CI	Specificity	95% CI	PPV	NPV	Youden's J
miR-155-3p	>0.1406	1.000	0.9324 to 1.000	0.042	0.002137 to 0.2024	1.000	1.000	0.042
	>0.1541	1.000	0.9324 to 1.000	0.083	0.01481 to 0.2585	1.000	1.000	0.083
	>0.2272	1.000	0.9324 to 1.000	0.125	0.04344 to 0.3100	1.000	1.000	0.125
	>0.3009	1.000	0.9324 to 1.000	0.167	0.06679 to 0.3585	1.000	1.000	0.167
	>0.3144	1.000	0.9324 to 1.000	0.208	0.09245 to 0.4047	1.000	1.000	0.208
	>0.3373	1.000	0.9324 to 1.000	0.250	0.1200 to 0.4490	1.000	1.000	0.250
	>0.3889	1.000	0.9324 to 1.000	0.292	0.1491 to 0.4917	1.000	1.000	0.292
	>0.4327	0.981	0.9006 to 0.9990	0.292	0.1491 to 0.4917	0.981	0.879	0.273
	>0.4623	0.981	0.9006 to 0.9990	0.333	0.1797 to 0.5329	0.981	0.892	0.314
	>0.4888	0.981	0.9006 to 0.9990	0.375	0.2116 to 0.5729	0.981	0.903	0.356
	>0.5065	0.962	0.8725 to 0.9933	0.375	0.2116 to 0.5729	0.962	0.824	0.337
	>0.5272	0.943	0.8463 to 0.9846	0.375	0.2116 to 0.5729	0.943	0.757	0.318
	>0.5433	0.925	0.8214 to 0.9703	0.375	0.2116 to 0.5729	0.925	0.700	0.300
	>0.5632	0.925	0.8214 to 0.9703	0.417	0.2447 to 0.6117	0.925	0.722	0.341
	>0.5968	0.925	0.8214 to 0.9703	0.458	0.2789 to 0.6493	0.925	0.741	0.383
	>0.6381	0.925	0.8214 to 0.9703	0.500	0.3143 to 0.6857	0.925	0.757	0.425
	>0.7685	0.925	0.8214 to 0.9703	0.542	0.3507 to 0.7211	0.925	0.772	0.466
	>0.8910	0.925	0.8214 to 0.9703	0.583	0.3883 to 0.7553	0.925	0.784	0.508

>0.9132	0.925	0.8214 to 0.9703	0.625	0.4271 to 0.7884	0.9250.796	0.550
>0.9406	0.906	0.7975 to 0.9590	0.625	0.4271 to 0.7884	0.9060.757	0.531
>0.9766	0.906	0.7975 to 0.9590	0.667	0.4671 to 0.8203	0.9060.769	0.572
>1.052	0.906	0.7975 to 0.9590	0.708	0.5083 to 0.8509	0.9060.779	0.614
>1.127	0.906	0.7975 to 0.9590	0.750	0.5510 to 0.8800	0.9060.789	0.656
>1.211	0.887	0.7742 to 0.9471	0.750	0.5510 to 0.8800	0.8870.757	0.637
>1.286	0.868	0.7516 to 0.9345	0.750	0.5510 to 0.8800	0.8680.728	0.618
>1.297	0.849	0.7295 to 0.9215	0.750	0.5510 to 0.8800	0.8490.701	0.599
>1.444	0.830	0.7077 to 0.9080	0.750	0.5510 to 0.8800	0.8300.675	0.580
>1.603	0.811	0.6864 to 0.8941	0.750	0.5510 to 0.8800	0.8110.652	0.561
>1.650	0.793	0.6654 to 0.8800	0.750	0.5510 to 0.8800	0.7930.630	0.543
>1.722	0.774	0.6447 to 0.8655	0.750	0.5510 to 0.8800	0.7740.609	0.524
>1.966	0.774	0.6447 to 0.8655	0.792	0.5953 to 0.9076	0.7740.622	0.565
>2.180	0.755	0.6243 to 0.8507	0.792	0.5953 to 0.9076	0.7550.603	0.546
>2.196	0.755	0.6243 to 0.8507	0.833	0.6415 to 0.9332	0.7550.615	0.588
>2.317	0.736	0.6042 to 0.8356	0.833	0.6415 to 0.9332	0.7360.597	0.569
>2.438	0.717	0.5843 to 0.8203	0.833	0.6415 to 0.9332	0.7170.581	0.550
>2.490	0.698	0.5646 to 0.8048	0.833	0.6415 to 0.9332	0.6980.565	0.531
>2.598	0.698	0.5646 to 0.8048	0.875	0.6900 to 0.9566	0.6980.577	0.573
>2.663	0.679	0.5452 to 0.7891	0.875	0.6900 to 0.9566	0.6790.562	0.554
>2.670	0.660	0.5259 to 0.7731	0.875	0.6900 to 0.9566	0.6600.548	0.535
>2.771	0.660	0.5259 to 0.7731	0.917	0.7415 to 0.9852	0.6600.560	0.577
>2.995	0.642	0.5069 to 0.7570	0.917	0.7415 to 0.9852	0.6420.546	0.558
>3.160	0.642	0.5069 to 0.7570	0.958	0.7976 to 0.9979	0.6420.557	0.600
>3.202	0.623	0.4881 to 0.7406	0.958	0.7976 to 0.9979	0.6230.544	0.581
>3.206	0.604	0.4694 to 0.7241	0.958	0.7976 to 0.9979	0.6040.532	0.562
>3.225	0.585	0.4509 to 0.7074	0.958	0.7976 to 0.9979	0.5850.521	0.543
>3.305	0.566	0.4327 to 0.6905	0.958	0.7976 to 0.9979	0.5660.510	0.524
>3.393	0.547	0.4145 to 0.6734	0.958	0.7976 to 0.9979	0.5470.499	0.506
>3.423	0.528	0.3966 to 0.6562	0.958	0.7976 to 0.9979	0.5280.489	0.487
>3.491	0.509	0.3788 to 0.6388	0.958	0.7976 to 0.9979	0.5090.479	0.468
>3.652	0.491	0.3612 to 0.6212	0.958	0.7976 to 0.9979	0.4910.470	0.449
>3.759	0.472	0.3438 to 0.6034	0.958	0.7976 to 0.9979	0.4720.461	0.430
>3.831	0.453	0.3266 to 0.5855	0.958	0.7976 to 0.9979	0.4530.452	0.411
>4.017	0.434	0.3095 to 0.5673	0.958	0.7976 to 0.9979	0.4340.443	0.392
>4.211	0.415	0.2926 to 0.5491	0.958	0.7976 to 0.9979	0.4150.435	0.373
>4.359	0.396	0.2759 to 0.5306	0.958	0.7976 to 0.9979	0.3960.428	0.355
>4.524	0.396	0.2759 to 0.5306	1.000	0.8620 to 1.000	0.3960.438	0.396
>4.617	0.377	0.2594 to 0.5119	1.000	0.8620 to 1.000	0.3770.430	0.377
>4.822	0.359	0.2430 to 0.4931	1.000	0.8620 to 1.000	0.3590.423	0.359
>5.038	0.340	0.2269 to 0.4741	1.000	0.8620 to 1.000	0.3400.416	0.340
>5.093	0.321	0.2109 to 0.4548	1.000	0.8620 to 1.000	0.3210.409	0.321
>5.164	0.302	0.1952 to 0.4354	1.000	0.8620 to 1.000	0.3020.403	0.302
>5.220	0.283	0.1797 to 0.4157	1.000	0.8620 to 1.000	0.2830.396	0.283
>5.469	0.264	0.1644 to 0.3958	1.000	0.8620 to 1.000	0.2640.390	0.264
>5.832	0.245	0.1493 to 0.3757	1.000	0.8620 to 1.000	0.2450.384	0.245
>6.126	0.226	0.1345 to 0.3553	1.000	0.8620 to 1.000	0.2260.378	0.226
>6.341	0.208	0.1200 to 0.3346	1.000	0.8620 to 1.000	0.2080.373	0.208
>6.470	0.189	0.1059 to 0.3136	1.000	0.8620 to 1.000	0.1890.367	0.189
>6.828	0.170	0.09200 to 0.2923	1.000	0.8620 to 1.000	0.1700.362	0.170
>7.427	0.151	0.07852 to 0.2705	1.000	0.8620 to 1.000	0.1510.357	0.151
>7.759	0.132	0.06548 to 0.2484	1.000	0.8620 to 1.000	0.1320.352	0.132
>7.989	0.113	0.05293 to 0.2258	1.000	0.8620 to 1.000	0.1130.347	0.113
>8.218	0.094	0.04097 to 0.2025	1.000	0.8620 to 1.000	0.0940.342	0.094
>8.279	0.075	0.02974 to 0.1786	1.000	0.8620 to 1.000	0.0750.337	0.075

	>8.588	0.057	0.01543 to 0.1537	1.000	0.8620 to 1.000	0.0570.333	0.057
	>8.883	0.038	0.006705 to 0.1275	1.000	0.8620 to 1.000	0.0380.328	0.038
	>9.276	0.019	0.0009678 to 0.09943	1.000	0.8620 to 1.000	0.0190.324	0.019
miR-3196	<0.055	0.021	0.001069 to 0.1090	1.000	0.8569 to 1.000	0.0210.325	0.021
	<0.059	0.042	0.007403 to 0.1398	1.000	0.8569 to 1.000	0.0420.329	0.042
	<0.064	0.063	0.02148 to 0.1684	1.000	0.8569 to 1.000	0.0630.334	0.063
	<0.066	0.083	0.03288 to 0.1955	1.000	0.8569 to 1.000	0.0830.339	0.083
	<0.070	0.104	0.04532 to 0.2217	1.000	0.8569 to 1.000	0.1040.344	0.104
	<0.073	0.125	0.05857 to 0.2470	1.000	0.8569 to 1.000	0.1250.350	0.125
	<0.080	0.167	0.08696 to 0.2958	1.000	0.8569 to 1.000	0.1670.361	0.167
	<0.087	0.188	0.1019 to 0.3194	1.000	0.8569 to 1.000	0.1880.367	0.188
	<0.089	0.208	0.1173 to 0.3426	1.000	0.8569 to 1.000	0.2080.373	0.208
	<0.096	0.229	0.1331 to 0.3654	1.000	0.8569 to 1.000	0.2290.379	0.229
	<0.106	0.250	0.1492 to 0.3878	1.000	0.8569 to 1.000	0.2500.386	0.250
	<0.120	0.271	0.1657 to 0.4100	1.000	0.8569 to 1.000	0.2710.392	0.271
	<0.131	0.292	0.1824 to 0.4318	1.000	0.8569 to 1.000	0.2920.399	0.292
	<0.137	0.313	0.1995 to 0.4533	1.000	0.8569 to 1.000	0.3130.407	0.313
	<0.141	0.333	0.2168 to 0.4746	1.000	0.8569 to 1.000	0.3330.414	0.333
	<0.142	0.354	0.2343 to 0.4956	1.000	0.8569 to 1.000	0.3540.421	0.354
	<0.145	0.375	0.2522 to 0.5164	1.000	0.8569 to 1.000	0.3750.430	0.375
	<0.149	0.396	0.2702 to 0.5369	1.000	0.8569 to 1.000	0.3960.438	0.396
	<0.156	0.417	0.2885 to 0.5572	1.000	0.8569 to 1.000	0.4170.447	0.417
	<0.167	0.438	0.3070 to 0.5772	1.000	0.8569 to 1.000	0.4380.456	0.438
	<0.179	0.458	0.3258 to 0.5971	1.000	0.8569 to 1.000	0.4580.465	0.458
	<0.188	0.479	0.3447 to 0.6167	1.000	0.8569 to 1.000	0.4790.475	0.479
	<0.215	0.500	0.3639 to 0.6361	1.000	0.8569 to 1.000	0.5000.485	0.500
	<0.271	0.521	0.3833 to 0.6553	1.000	0.8569 to 1.000	0.5210.496	0.521
	<0.328	0.542	0.4029 to 0.6742	1.000	0.8569 to 1.000	0.5420.507	0.542
	<0.355	0.563	0.4228 to 0.6930	1.000	0.8569 to 1.000	0.5630.519	0.563
	<0.366	0.583	0.4428 to 0.7115	1.000	0.8569 to 1.000	0.5830.530	0.583
	<0.391	0.583	0.4428 to 0.7115	0.957	0.7901 to 0.9978	0.5830.519	0.540
	<0.426	0.604	0.4631 to 0.7298	0.957	0.7901 to 0.9978	0.6040.532	0.561
	<0.449	0.625	0.4836 to 0.7478	0.957	0.7901 to 0.9978	0.6250.546	0.582
	<0.452	0.625	0.4836 to 0.7478	0.913	0.7320 to 0.9845	0.6250.534	0.538
	<0.461	0.625	0.4836 to 0.7478	0.870	0.6787 to 0.9546	0.6250.522	0.495
	<0.471	0.625	0.4836 to 0.7478	0.826	0.6286 to 0.9302	0.6250.509	0.451
	<0.494	0.646	0.5044 to 0.7657	0.826	0.6286 to 0.9302	0.6460.523	0.472
	<0.545	0.646	0.5044 to 0.7657	0.783	0.5810 to 0.9034	0.6460.510	0.429
	<0.581	0.667	0.5254 to 0.7832	0.783	0.5810 to 0.9034	0.6670.525	0.450
	<0.592	0.667	0.5254 to 0.7832	0.739	0.5353 to 0.8745	0.6670.511	0.406
	<0.606	0.667	0.5254 to 0.7832	0.696	0.4913 to 0.8440	0.6670.496	0.363
	<0.647	0.688	0.5467 to 0.8005	0.696	0.4913 to 0.8440	0.6880.512	0.384
	<0.682	0.688	0.5467 to 0.8005	0.652	0.4489 to 0.8119	0.6880.496	0.340
	<0.709	0.688	0.5467 to 0.8005	0.609	0.4079 to 0.7784	0.6880.479	0.297
	<0.751	0.688	0.5467 to 0.8005	0.565	0.3681 to 0.7437	0.6880.460	0.253
	<0.781	0.688	0.5467 to 0.8005	0.522	0.3296 to 0.7076	0.6880.441	0.210
	<0.803	0.688	0.5467 to 0.8005	0.478	0.2924 to 0.6704	0.6880.419	0.166
	<0.813	0.708	0.5682 to 0.8176	0.478	0.2924 to 0.6704	0.7080.435	0.186
	<0.829	0.708	0.5682 to 0.8176	0.435	0.2563 to 0.6319	0.7080.412	0.143
	<0.847	0.729	0.5900 to 0.8343	0.435	0.2563 to 0.6319	0.7290.430	0.164
	<0.866	0.729	0.5900 to 0.8343	0.391	0.2216 to 0.5921	0.7290.404	0.120
	<0.895	0.750	0.6122 to 0.8508	0.391	0.2216 to 0.5921	0.7500.424	0.141
	<0.908	0.750	0.6122 to 0.8508	0.348	0.1881 to 0.5511	0.7500.396	0.098
	<0.911	0.771	0.6346 to 0.8669	0.348	0.1881 to 0.5511	0.7710.417	0.119
	<0.928	0.792	0.6574 to 0.8827	0.348	0.1881 to 0.5511	0.7920.441	0.140

<0.946	0.813	0.6806 to 0.8981	0.348	0.1881 to 0.5511	0.8130.467	0.161
<1.11	0.833	0.7042 to 0.9130	0.348	0.1881 to 0.5511	0.8330.495	0.181
<1.28	0.854	0.7283 to 0.9275	0.348	0.1881 to 0.5511	0.8540.529	0.202
<1.30	0.875	0.7530 to 0.9414	0.348	0.1881 to 0.5511	0.8750.567	0.223
<1.32	0.896	0.7783 to 0.9547	0.348	0.1881 to 0.5511	0.8960.612	0.244
<1.34	0.917	0.8045 to 0.9671	0.348	0.1881 to 0.5511	0.9170.664	0.265
<1.38	0.938	0.8316 to 0.9785	0.348	0.1881 to 0.5511	0.9380.725	0.286
<1.41	0.958	0.8602 to 0.9926	0.348	0.1881 to 0.5511	0.9580.796	0.306
<1.44	0.958	0.8602 to 0.9926	0.304	0.1560 to 0.5087	0.9580.773	0.262
<1.46	0.979	0.8910 to 0.9989	0.304	0.1560 to 0.5087	0.9790.872	0.283
<1.48	1.000	0.9259 to 1.000	0.304	0.1560 to 0.5087	1.0001.000	0.304
<1.50	1.000	0.9259 to 1.000	0.261	0.1255 to 0.4647	1.0001.000	0.261
<1.58	1.000	0.9259 to 1.000	0.217	0.09664 to 0.4190	1.0001.000	0.217
<1.70	1.000	0.9259 to 1.000	0.174	0.06979 to 0.3714	1.0001.000	0.174
<2.03	1.000	0.9259 to 1.000	0.130	0.04538 to 0.3213	1.0001.000	0.130
<2.36	1.000	0.9259 to 1.000	0.087	0.01545 to 0.2680	1.0001.000	0.087
<3.15	1.000	0.9259 to 1.000	0.043	0.002230 to 0.2099	1.0001.000	0.044

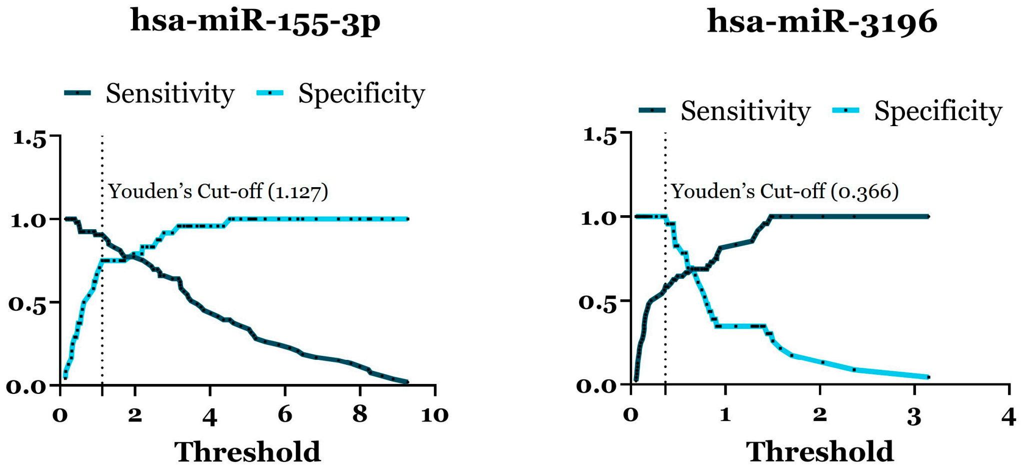

Figure S2. Sensitivity and specificity as a function of the decision threshold for PBMC miR-155-3p and miR-3196. The dotted lines denotes the Youden-optimized ΔΔCt threshold (maximum J).

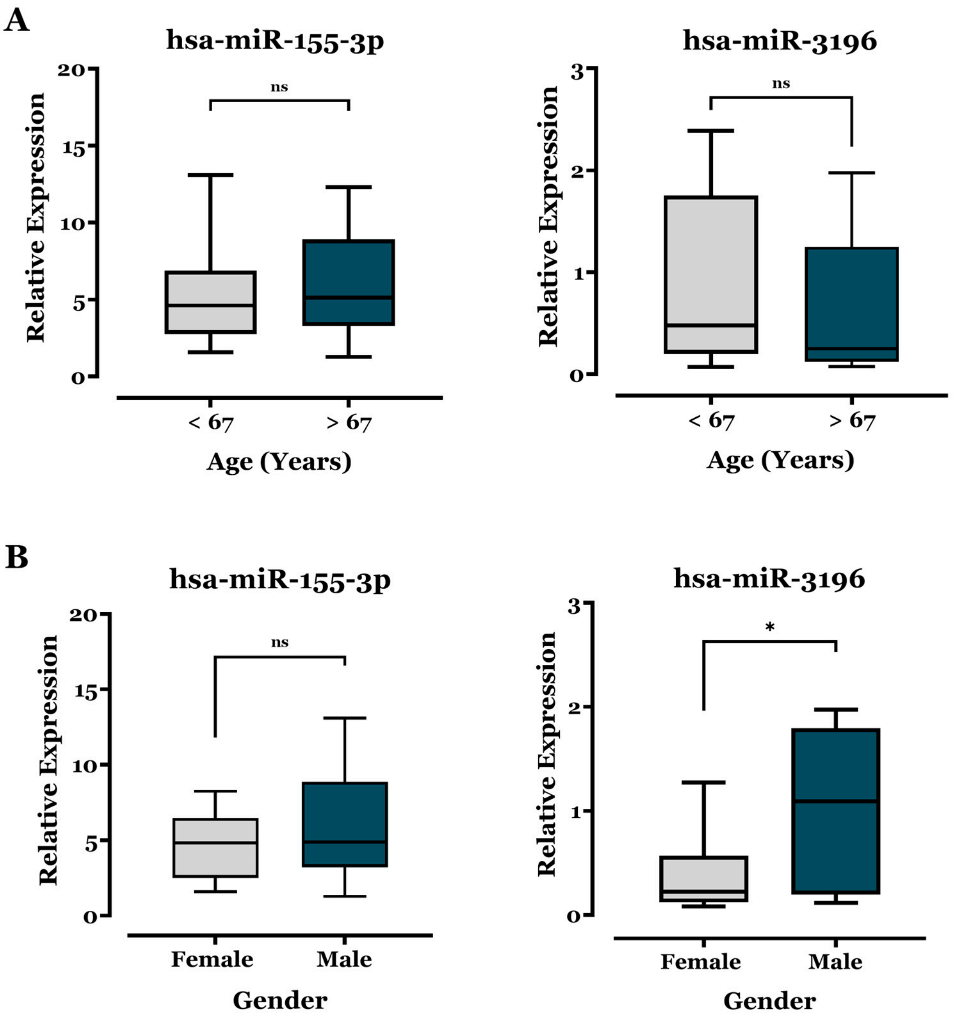

Figure S3. Comparison of miR-155-3p and miR-3196 expression in different subgroups of ADC patients. (**A**) RT–qPCR analysis of differential of miRs levels in different ages of patients. The expression levels of miRs were normalized relative to the corresponding expression level of U6 snRNA and relative expression was determined using the $2^{-\Delta\Delta Ct}$ method. Mann–Whitney test was used to determine the significance of the association and values are expressed in means ± SEM and displayed on a box plot. "ns": non-significant. (**B**) RT–qPCR analysis of miRs levels in two genders of patients. The expression levels of miRs were normalized relative to the corresponding expression level of U6 snRNA and relative expression was determined using the $2^{-\Delta\Delta Ct}$ method. Mann–Whitney test was used to determine the significance of the association and values are expressed in means ± SEM and displayed on a box plot. * $\rho < 0.05$ and "ns": non-significant.

Table S2. The comparison between miR-155-3p and miR-3196 levels and clinicopathological parameters in NSCLC patients. Variations in sample numbers (*n*) across variables reflect missing clinical information in the medical records and do not indicate loss or failure of PBMC miR analysis, except for a small subset of samples whose miR expression results were excluded due to technical unreliability.

Clinicopathologic Parameters	miR-155-3p ($2^{-\Delta\Delta Ct}$)			miR-3196 ($2^{-\Delta\Delta Ct}$)		
	n	Mean ± SEM	*ρ*-value	*n*	Mean ± SEM	*ρ*-value
Clinical Stage						
Early (I–II)	7	3.69 ± 0.46	0.0084	6	0.91 ± 0.40	0.2768
Advanced (III–IV)	34	5.90 ± 0.64		23	0.41 ± 0.09	
Metastasis						
M0	11	3.13 ± 0.54	0.0450	7	1.17 ± 0.33	0.0637

M1a	11	4.72 ± 1.01	(M0 vs. M1c)	7	0.38 ± 0.14	(M0 vs. M1c)
M1b	6	5.82 ± 1.63		4	0.31 ± 0.16	
M1c	13	6.12 ± 1.01		9	0.21 ± 0.05	
Age (years)						
≤67	21	5.11 ± 0.68	0.4697	13	0.84 ± 0.22	0.5060
>67	21	5.88 ± 0.75		21	0.70 ± 0.15	
Gender						
Female	12	4.66 ± 0.68	0.4829	17	0.41 ± 0.11	0.0328
Male	30	5.83 ± 0.65		15	1.03 ± 0.19	
Smoking Status						
Never	10	2.58 ± 0.25	<0.0001 (Never vs. Current)	11	0.60 ± 0.20	0.4550 (Never vs. Current)
Former	11	5.11 ± 0.94		9	1.18 ± 0.29	
Current	16	7.70 ± 0.73		9	0.36 ± 0.08	
Gene Mutations						
Negative	19	6.93 ± 0.82	0.3713 (Neg. vs. EGFR) 0.1246 (Neg. vs. KRAS)	11	0.63 ± 0.22	>0.9999
ALK	3	5.31 ± 1.62		3	0.94 ± 0.46	
BRAF	3	6.37 ± 2.71		2	1.05 ± 0.05	
EGFR	10	4.08 ± 0.56		12	0.77 ± 0.20	
KRAS	4	2.84 ± 0.89		3	0.69 ± 0.55	
PIK3CA	3	6.55 ± 2.86		3	0.17 ± 0.05	
ROS-1	3	3.99 ± 1.32		2	0.24 ± 0.17	
RET	1	0.42 ± 0.00		1	0.08 ± 0.00	
MET	1	1.46 ± 0.00		1	0.03 ± 0.00	

n number of individuals. **SEM** standard error of the mean.
